# Pnserpin: A Novel Serine Protease Inhibitor from Extremophile *Pyrobaculum neutrophilum*

**DOI:** 10.3390/ijms18010113

**Published:** 2017-01-07

**Authors:** Huan Zhang, Rui Fei, Baigong Xue, Shanshan Yu, Zuoming Zhang, Sheng Zhong, Yuanqi Gao, Xiaoli Zhou

**Affiliations:** 1 Department of Cell Biology, College of Basic Medical Sciences, Jilin University, Changchun 130021, China; zhanghuan1990@yahoo.com (H.Z.); feirui@jlu.edu.cn (R.F.); xuebg@jlu.edu.cn (B.X.); zhongsheng2016@aliyun.com (S.Z.); gaoyq@jlu.edu.cn (Y.G.); 2 State Key Laboratory of Microbial Metabolism, School of Life Sciences and Biotechnology, Shanghai Jiao Tong University, Shanghai 200240, China; yushanshan001@aliyun.com; 3 Key Laboratory for Molecular Enzymology and Engineering, Ministry of Education, Jilin University, Changchun 130012, China; zmzhang@jlu.edu.cn

**Keywords:** Pnserpin, serine protease inhibitor, thermophile

## Abstract

Serine protease inhibitors (serpins) are native inhibitors of serine proteases, constituting a large protein family with members spread over eukaryotes and prokaryotes. However, only very few prokaryotic serpins, especially from extremophiles, have been characterized to date. In this study, Pnserpin, a putative serine protease inhibitor from the thermophile *Pyrobaculum neutrophilum*, was overexpressed in *Escherichia coli* for purification and characterization. It irreversibly inhibits chymotrypsin-, trypsin-, elastase-, and subtilisin-like proteases in a temperature range from 20 to 100 °C in a concentration-dependent manner. The stoichiometry of inhibition (SI) of Pnserpin for proteases decreases as the temperature increases, indicating that the inhibitory activity of Pnserpin increases with the temperature. SDS-PAGE (sodium dodecyl sulfate polyacrylamide gel electrophoresis) showed that Pnserpin inhibits proteases by forming a SDS-resistant covalent complex. Homology modeling and molecular dynamic simulations predicted that Pnserpin can form a stable common serpin fold. Results of the present work will help in understanding the structural and functional characteristics of thermophilic serpin and will broaden the current knowledge about serpins from extremophiles.

## 1. Introduction

Serine protease inhibitors (serpins) comprise a superfamily of proteins with inhibitory activity towards serine or cysteine proteases. To date, more than 1500 members of this superfamily have been found, and they are widely distributed in eukaryotes, bacteria, and archea [1,2]. By inhibiting specific proteases, serpins, such as antithrombin [3], α1-antitrypsin [4,5], and neuroserpin [6,7], are involved in many important biological processes, including inflammation, blood coagulation, and fibrinolysis. Some other members with non-inhibitory activities may behave as chaperones or transport molecules, and participate in cellular processes [8,9,10].

The average molecular size of serpin proteins is 350–400 amino acids, with molecular weights of approximately 40–50 kD [11]. Members of the serpin family adopt a conserved structure. Generally, their structures contain three β sheets (A, B, C), seven to nine α helixes (hA–hZ) and a protease recognition site termed the reactive center loop (RCL). The RCL is an exposed stretch of residues, typically consisting of 20–24 residues, which displays a high conformational flexibility. This loop is related to the function of serpins, responsible for the initial binding of serpin with protease and the forming of a non-covalent complex.

Serpins are suicide inhibitors of protease, relying on a complex conformational change to inhibit the target protease. The interaction between RCL and protease triggers a major conformational change, where the cleaved RCL is inserted into the middle of the five-stranded β-sheet A to form a six-stranded anti-parallel β-sheet at the core of the serpin structure, a process referred to as the stressed-relaxed (S to R) transition. This conformational rearrangement drags the protease to the opposite side and leads to its inactivation, resulting in a covalent serpin-protease complex that is highly resistant to thermal and chemical denaturation [12,13,14]. Thus, the structural metastability of serpin is essential to its inhibitory activity and biological function.

The serpin genes have also been found in thermophiles in recent years. To date, only very few of them have been biochemically and structurally characterized, such as thermopin from the moderate thermophilic bacterium *Thermobifida fusca* (with an optimum growth temperature of 55 °C) [15,16], tengpin from the thermophilic bacterium *Thermoanaerobactor tengcondensis* (with an optimum growth temperature of 75 °C) [17,18], Tk-serpin from the hyperthermophilic archaeon *Thermococcus kodakaraensis* (with an optimum growth temperature of 90 °C) [19], and aeropin from the hyperthermophilic archaeon *Pyrobaculum aerophilum* (with an optimum growth temperature over 100 °C) [20]. We are interested in the fact that these serpins show inhibitory activities toward serine proteases, even at such high temperatures. Structural information of thermopin indicated that a C-terminal tail plays an important role in its folding and function [15,16]. Moreover, the study of tengpin showed that a hydrophobic patch formed by the N-terminus amino acids is essential to its conformational change [17,18]. Other studies on thermophilic serpins have also indicated the importance of multiple salt bridges, hydrogen bonds, hydrophobic interactions and cation-pi interactions to the stability of the structure at high temperatures while maintaining their function in inhibiting proteases [19,20]. There are still many serpins that exist in extremophile genomes that are uncharacterized. Their structures and functions need to be investigated in order to understand their specific mechanisms.

*Pyrobaculum neutrophilum* is a hyperthermophilic archaeon and grows most optimally at 80 °C [21]. Its genome contains a gene encoding for a serpin homologue (GenBank: ACB40836.1), which we named Pnserpin. The structure and function of Pnserpin has not been reported. In the present study, we cloned and overexpressed Pnserpin in *E. coli*. After purification, its inhibitory activity towards serine proteases and the temperature and pH stabilities of Pnserpin were characterized. Furthermore, homology modeling and molecular dynamic simulations were used to analyze the probable structure of Pnserpin. Our results showed that Pnserpin is a functional serpin with high temperature and pH stabilities.

## 2. Results

### 2.1. Sequence Analysis of Pnserpin

**Figure 1:**
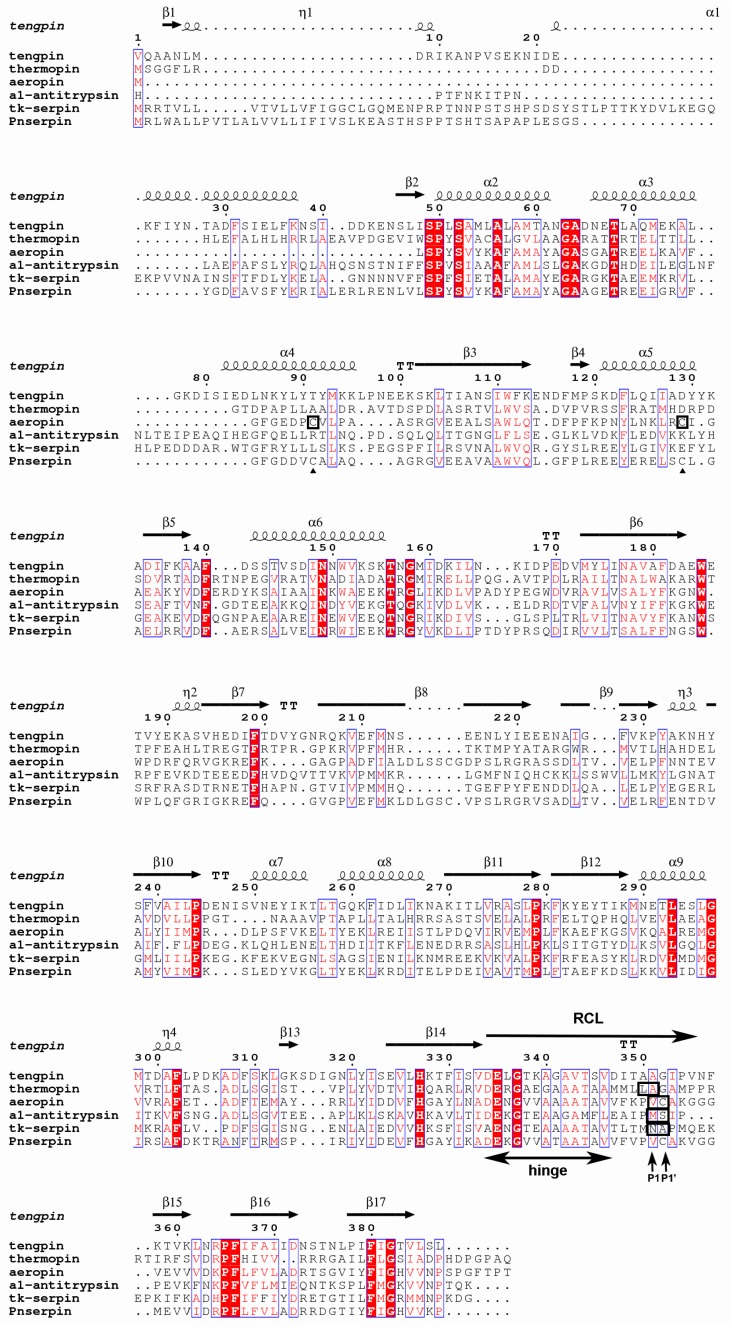
Multiple sequence alignment of serpin sequences. The amino acid sequence of Pnserpin is compared with those of α1-antitrypsin, thermopin, aeropin, tengpin, and Tk-serpin. The highly conserved amino acid residues, which are conserved in >70% of the members of the serpin superfamily, are highlighted in red letters. The residues conserved in 100% are highlighted in red background. The conserved hinge region, the possible P1 and P1’ sites and the reactive center loop (RCL) region are indicated by arrows. The P1–P1’ positions in RCL, which have been reported, are in black boxes. The Cys102–Cys136 of aeropin is in black boxes, and the corresponding residues of Pnserpin are indicated by a triangle. The figure was produced using ESPript3.0 [23].

In the genome sequence data of the thermophilic archaeon *Pyrobaculum neutrophilum*, a gene coding for a serine protease inhibitor (Pnserpin, GenBank: ACB40836.1), which is composed of 393 amino acid residues, was found. Sequence alignment of Pnserpin with other serpins (Figure 1) demonstrated that it possesses most of the highly conserved residues of the serpin superfamily, suggesting that it conforms to the common serpin structure. Pnserpin shares a 51% amino acid sequence identity with aeropin from the extremophile *Pyrobaculum aerophilum*, and it shares a lower sequence identity with other serpins. The sequence alignment also indicates that Pnserpin is likely to be a protease inhibitor due to the presence of an alanine repeat motif (amino acids 346–351, ATAATA) in the hinge region which commonly exists in inhibitory serpins [22]. Despite low levels of overall amino acid sequence identity, the residues in the hinge region of the RCL are highly conservative, especially among these thermophilic serpins (Pnserpin, Tk-serpin, aeropin, thermopin and tengpin). According to the multiple sequence alignment, the cleavage sites of aeropin, α1-antitrypsin, and Tk-serpin correspond to Val357–Cys358 of Pnserpin. Therefore, we speculate that the P1 and P1’ sites of Pnserpin may be Val357 and Cys358 in the reactive center loop.

### 2.2. Cloning, Expression, and Purification of Pnserpin

**Figure 2:**
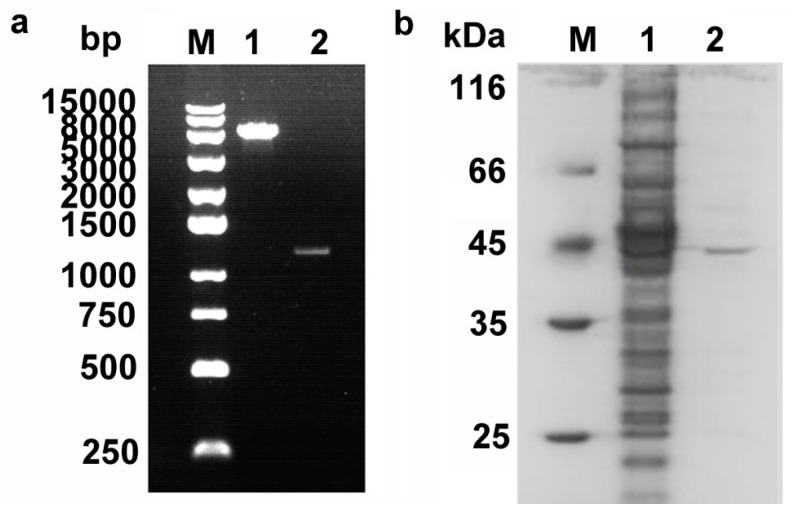
Cloning and purification of Pnserpin. (**a**) Cloning of the Pnserpin gene. Lane M, marker 250 bp DNA ladder; Lane 1, pET-28a(+); Lane 2, full-length DNA fragment of Pnserpin; (**b**) Purification of recombinant protein Pnserpin. Lane M, molecular mass marker; Lane 1, the crude extract; Lane 2, purified Pnserpin after Ni^2+^ affinity chromatography.

To investigate whether Pnserpin is an inhibitory serpin, the gene of Pnserpin was spliced and cloned in the pET-28a(+) expression vector (Figure 2a). Soluble recombinant N-terminally His6-tagged Pnserpin was produced in *E. coli* BL21-CodonPlus (DE3)-RIL and purified by nickel-chelating chromatography. As shown in Figure 2b, the purified Pnserpin protein showed a single band with a molecular mass of 44 kDa.

### 2.3. Inhibition of Proteases by Pnserpin

**Figure 3:**
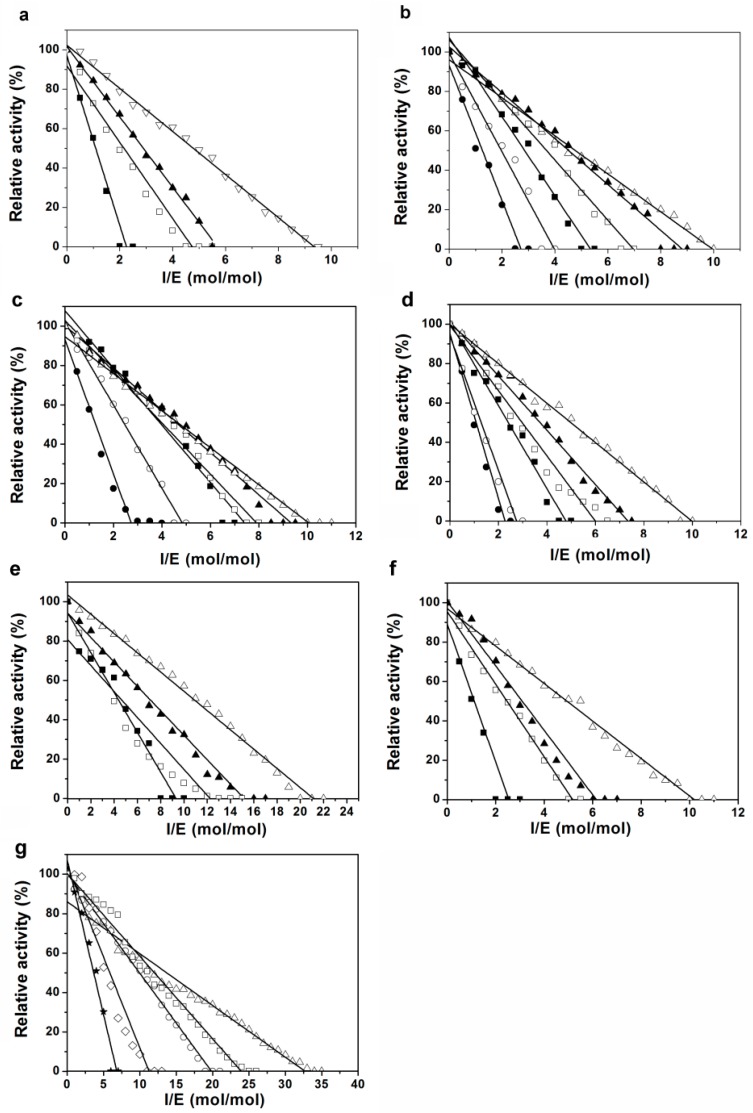
Effect of Pnserpin:protease molar ratio and temperature on protease inhibition by Pnserpin. CHT (**a**), SUC (**b**), elastase (**c**), PRK (**d**), thrombin (**e**), trypsin (**f**), and PnCHT (**g**) were incubated with Pnserpin (inhibitor) at various molar ratios at 20 °C (∆), 30 °C (▲), 40 °C (□), 50 °C (■), 60 °C (○), 70 °C (●), 80 °C (◇) and 100 °C (★).

To examine whether Pnserpin exhibits inhibitory activity for various proteases and to determine the stoichiometry of the inhibition (SI) values of Pnserpin for these proteases, bovine α-chymotrypsin (CHT), subtilisin Carlsberg (SUC), porcine pancreatic elastase, proteinase k (PRK), bovine plasma thrombin, and bovine pancreatic trypsin were incubated with Pnserpin at various molar ratios and their residual activities were determined at 25 °C. The incubation temperature range was from 20 to 70 °C for SUC, elastase, and PRK, and 20 to 50 °C for CHT, thrombin, and trypsin as these enzymes are not stable at temperatures above 50 °C. As shown in Figure 3, all the proteases were inhibited in a concentration-dependent manner in the determined temperature range, indicating that Pnserpin can inhibit these proteases. The SI values of Pnserpin for these proteases are listed in Table 1. For all the proteases we tested, the SI value of Pnserpin decreased as the temperature increased. This result is similar to that of Tk-serpin [19], indicating that the inhibitory activity of Pnserpin increases as the temperature increases.

The genome of *Pyrobaculum neutrophilum* contains a gene encoding chymotrypsin-like serine protease (GenBank: ACB40794.1), which we named PnCHT. To determine whether Pnserpin exhibits inhibitory activity for PnCHT, we cloned the gene of PnCHT and expressed and purified the protein from *E. coli.* The purified PnCHT was incubated with Pnserpin at various molar ratios in the temperature range from 20 to 100 °C and the residual activities were determined at 40 °C. As shown in Figure 3g and Table 1, the SI values of Pnserpin for PnCHT also exhibited a temperature-dependent manner. At 20–60 °C, the SI values of Pnserpin for PnCHT were higher, indicating lower inhibitory activities, while at 80 and 100 °C, the SI values decreased to 11.26 and 6.82, indicating higher inhibitory activities. These results demonstrated that Pnserpin can inhibit PnCHT in a wide range of temperatures (20–100 °C).

### 2.4. Association Rate Constant of Pnserpin

**Figure 4:**
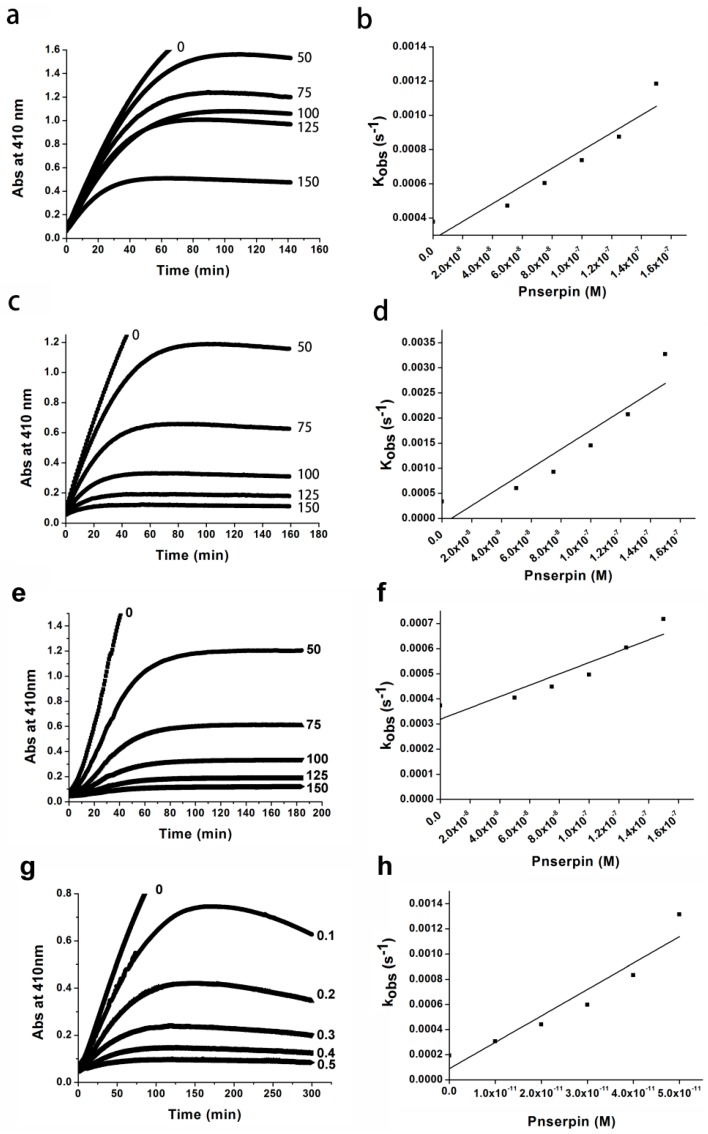

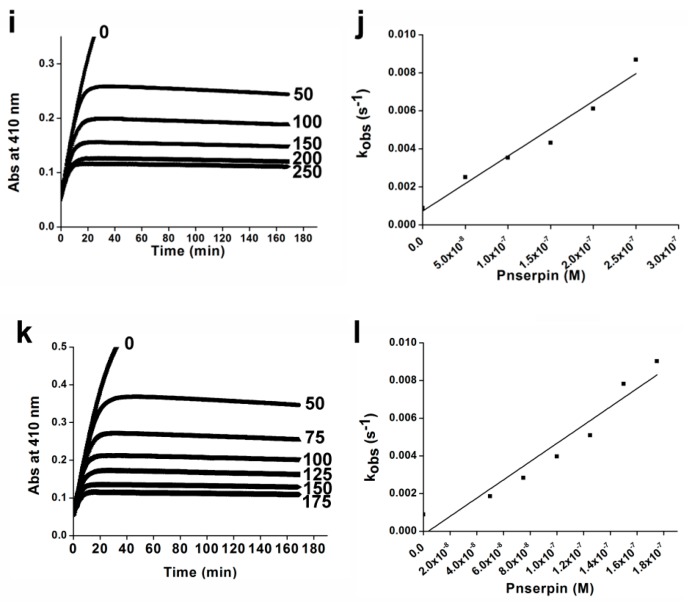
Progress curve analysis of protease inhibition by Pnserpin. (**a**,**c**,**e**,**g**,**i**) Progress curves for inhibition of CHT, SUC, elastase, PRK, and PnCHT by Pnserpin at 40 °C, respectively; (**k**) Progress curve for inhibition of PnCHT at 80 °C; (**b**,**d**,**f**,**h**,**j**) Plots of the *k*_obs_ values of CHT, SUC, elastase, PRK, and PnCHT as a function of Pnserpin concentration at 40 °C, respectively; (**l**) Plot of the *k*_obs_ values of PnCHT as a function of Pnserpin concentration at 80 °C. The number associated with each progress curve represents the concentration of Pnserpin (nM).

The second-order *k*_ass_ values of Pnserpin for the inhibition of CHT, SUC, elastase, PRK and PnCHT were determined by the progress curve method. The enzymatic activities of these proteases were analyzed in the presence of various concentrations of Pnserpin, using suc-AAPF-*p*NA as a substrate for CHT, SUC, PRK, and PnCHT, and MeO-SucAAPV-*p*NA as a substrate for elastase. The progress curves were obtained by monitoring the increased absorption at 410 nm, which results from the release of *p*-nitroaniline. These curves are hyperbolic curves, and the plot of the *k*_obs_ value as a function of Pnserpin concentration indicates a linear relationship between them (Figure 4). The resulting *k*_ass_ values of Pnserpin for CHT, SUC, elastase, and PRK were 2.46 × 10^4^, 1.30 × 10^5^, 2.27 × 10^4^ and 1.98 × 10^5^ M^−1^·s^−1^, respectively. The *k*_ass_ values of Pnserpin for PnCHT at 40 and 80 °C were 3.9 × 10^4^ and 1.07 × 10^5^ M^−1^·s^−1^, respectively. These results show that Pnserpin interacts with proteases with different association rate constants; Pnserpin showed a greater inhibitory effect on SUC and PRK than CHT, PnCHT and elastase at lower temperatures. Moreover, the inhibitory effect was stronger at higher temperatures.

### 2.5. The Temperature and pH Stabilities of Pnserpin

**Figure 5:**
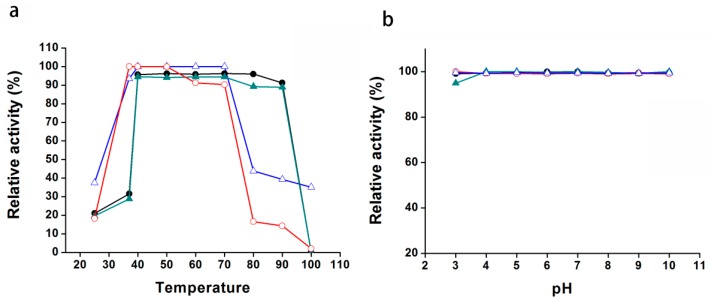
The temperature and pH stabilities of Pnserpin. (**a**) Effects of temperature on the activity of Pnserpin. The inhibitor was incubated at the indicated temperature for 15 min, and the residual inhibitory activities against CHT (red open circle), SUC (blue open triangle), elastase (black solid circle), and PRK (green solid triangle) were measured; (**b**) Effects of pH on the activity of Pnserpin. The inhibitor was incubated at the indicated pH for 24 h at room temperature, and the residual inhibitory activities against CHT (red open circle), SUC (blue open triangle), elastase (black solid circle), and PRK (green solid triangle) were measured.

In general, proteins from thermophiles are highly stable. To investigate the temperature stability of Pnserpin, we measured the remaining inhibitory activity of Pnserpin after being incubated in different temperature ranges from 25 to 100 °C for 15 min. As shown in Figure 5a, after incubation, Pnserpin was active across this temperature range. More than 90% inhibitory activity remained after incubation at temperatures from 35 to 75 °C. At temperatures below 30 °C or above 80 °C, the activity decreased rapidly. However, even when incubated at 100 °C for 15 min, Pnserpin still maintained a 40% inhibitory activity for SUC.

We assayed the effect of pH changes on the inhibitory activity of Pnserpin in the range of pH 3–10. The results showed that, in the entire measured range, Pnserpin maintained nearly 100% inhibitory activity for all the tested proteases (Figure 5b). The above results indicated that Pnserpin remained stable across the temperature range of 25 to 100 °C and the pH range of 3–10.

### 2.6. Formation of Covalent Complex between Pnserpin and Target Proteases

**Figure 6:**
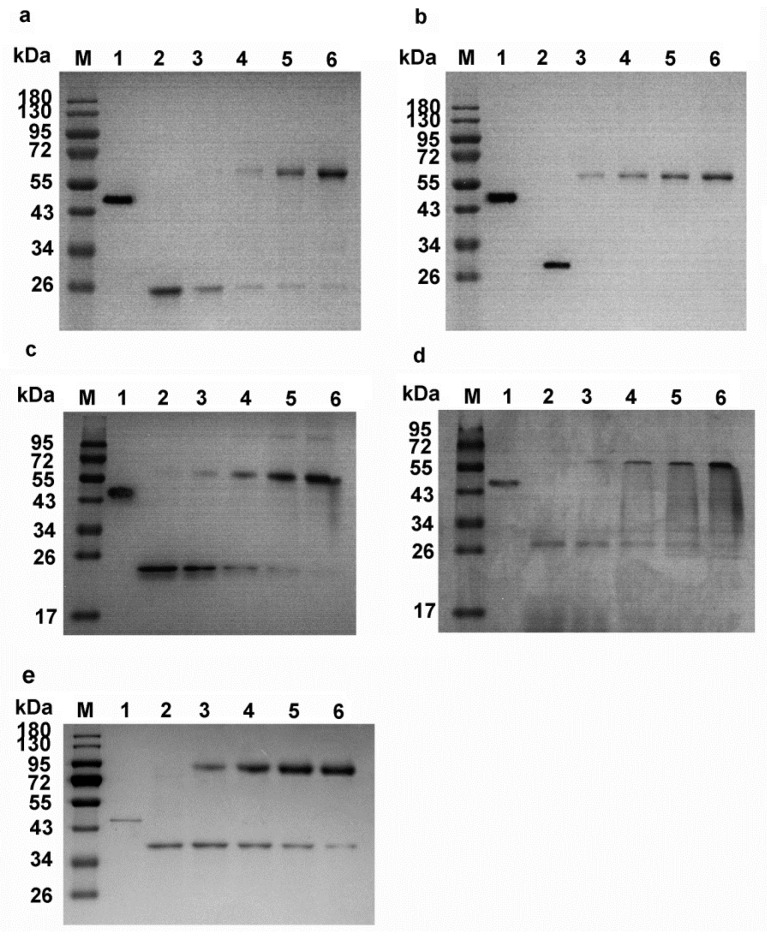
SDS-PAGE analysis of stable covalent complexes of Pnserpin with serine proteases. (**a**) CHT incubated with increasing concentrations of Pnserpin. Lane M, molecular mass marker; Lane 1, Pnserpin alone; Lane 2, CHT alone; Lane 3, Pnserpin/CHT = 0.5; Lane 4, Pnserpin/CHT = 1; Lane 5, Pnserpin/CHT = 2; Lane 6, Pnserpin/CHT = 4; (**b**) SUC incubated with increasing concentrations of Pnserpin. Lane M, molecular mass marker; Lane 1, Pnserpin alone; Lane 2, SUC alone; Lane 3, Pnserpin/SUC = 0.5; Lane 4, Pnserpin/SUC = 1; Lane 5, Pnserpin/SUC = 2; Lane 6, Pnserpin/SUC = 4; (**c**) Elastase incubated with increasing concentrations of Pnserpin. Lane M, molecular mass marker; Lane 1, Pnserpin alone; Lane 2, elastase alone; Lane 3, Pnserpin/elastase = 0.5; Lane 4, Pnserpin/elastase = 1; Lane 5, Pnserpin/elastase = 2; Lane 6, Pnserpin/elastase = 4; (**d**) PRK incubated with increasing concentrations of Pnserpin. Lane M, molecular mass marker; Lane 1, Pnserpin alone; Lane 2, PRK alone; Lane 3, Pnserpin/PRK = 0.5; Lane 4, Pnserpin/PRK = 1; Lane 5, Pnserpin/PRK = 2; Lane 6, Pnserpin/PRK = 4; (**e**) PnCHT incubated with increasing concentrations of Pnserpin. Lane M, molecular mass marker; Lane 1, Pnserpin alone; Lane 2, PnCHT alone; Lane 3, Pnserpin/PnCHT = 0.5; Lane 4, Pnserpin/PnCHT = 1; Lane 5, Pnserpin/PnCHT = 2; Lane 6, Pnserpin/PnCHT = 4.

To examine whether Pnserpin forms a covalent complex with target protease, as other serpins do, a constant amount of proteases was incubated with increasing amounts of Pnserpin. The reaction was stopped by boiling in reducing SDS-PAGE (sodium dodecyl sulfate polyacrylamide gel electrophoresis) sample buffer, and the mixtures were analyzed using SDS-PAGE (Figure 6). This analysis revealed the presence of a band between 55–72 kD or a band between 72–95 kD. As mentioned above, according to the multiple sequence alignment, the P1 and P1’ sites of Pnserpin might be Val357 and Cys358 in the reactive center loop. Therefore, we hypothesized that the molecular weight of the complex formed by Pnserpin with CHT, SUC, elastase, PRK, and PnCHT could be ~65, ~67, ~64, ~68, and ~76 kD, respectively (the molecular weights of CHT, SUC , elastase and PRK are 25, 27, 24, 28, and 36 kD, respectively). These predicted molecular weights of the complexes are consistent with the results from the SDS-PAGE analysis of the covalent complexes.

### 2.7. Homology Modeling and Molecular Dynamic Simulation

**Figure 7:**
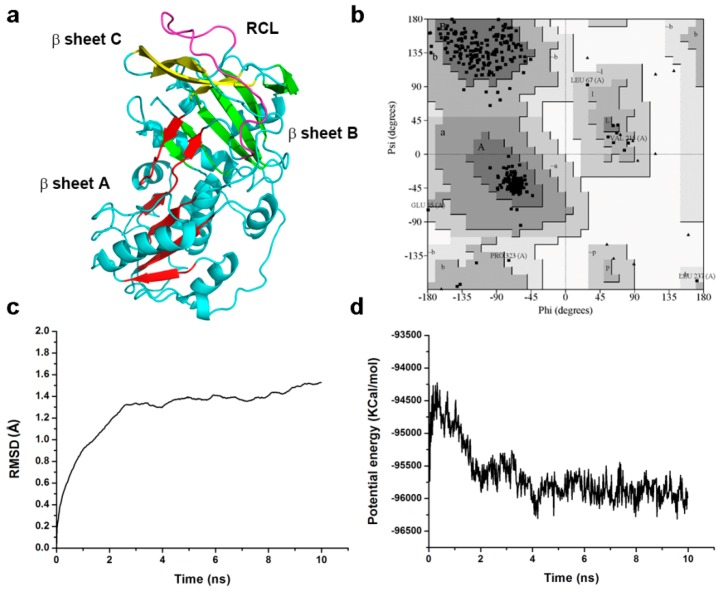
Homology modeling and molecular dynamics simulation of Pnserpin. (**a**) Model structure of Pnserpin. β sheet A, β sheet B and β sheet C are colored in red, green, and yellow, respectively; the reactive center loop (RCL) is colored in purple; (**b**) The Ramachandran plot of the model structure of Pnserpin. Dark grey, most favored region; grey, allowed region; light grey, generously allowed region. The residue numbers show the residues in the generously allowed region; (**c**,**d**) Average backbone RMSD and potential energy of 10 ns molecular dynamic simulation of the Pnserpin model structure.

The structures of tengpin (PDB ID: 2PEE) [17] from the thermophile *Caldanaerobacter subterraneus* and IRS-2 (PDB ID: 3NDA) [24] from *Ixodes ricinus* were selected as templates to build a 3D model of the structure of Pnserpin. The sequence identities are 20.1% for Pnserpin and tengpin and 26.6% for Pnserpin and IRS-2. We constructed the structural model of Pnserpin using Discovery Studio3.5 (DS3.5, Accelrys, Inc., San Diego, CA, USA). The observed 3D structure of Pnserpin shows the presence of nine α-helices, three β-sheets, and an exposed mobile reactive center loop (RCL: amino acids 345–365) (Figure 7a). The quality of the model was evaluated using the PROCHECK [25] and Profile_3D programs [26,27]. The results indicated that more than 92% of the residues in the model were located in the most favored region of the Ramachandran plot, and none of the residues were located in the disallowed region (Figure 7b). The Profile_3D verify score was 169.8, close to the expected high score of 170.9 and much higher than the expected low score of 76.9 (Table 2), indicating the good quality of the model structure. To evaluate the stabilities of the model structure under dynamic conditions, we conducted molecular dynamics (MD) simulations using DS3.5. The RMSD (root-mean-square deviation) curves of the backbone and the potential energy profiles are shown in Figure 7c,d. The trajectories of the model reached equilibrium after 4 ns, and the RMSD and potential energy stabilized with time. These results indicated that Pnserpin can form a stable common serpin fold, and confirmed that Pnserpin is a member of the serpin superfamily.

## 3. Discussion

In this report, we showed that Pnserpin from the thermophilic archaeon *Pyrobaculum neutrophilum* is functional in a wide range of temperatures and pH values. One important parameter that describes the efficiency of serpins as inhibitors is the SI value, which represents the number of serpin molecules required to inhibit one molecule of a target protease. Similar to other serpins from thermopiles, such as Tk-serpin, the SI values of Pnserpin for proteases decreased as the temperature increased. The SI values of Pnserpin for CHT, SUC, PRK, and PnCHT were close to those of Tk-serpin at higher temperatures (60–100 °C), while at lower temperatures (20–50 °C), the SI values of Pnserpin were lower than those of Tk-serpin. This may be because the flexibilities of the serpins are higher when the temperature approaches the optimum growth temperature of their source organism (80 °C for *Pyrobaculum neutrophilum* and 90 °C for *Thermococcus kodakaraensis*), and the conformations of the serpins are more favorable to the inhibitory activity. However, in a low temperature range, the difference in flexibility leads to a difference in the activity of serpins.

The inhibitory activity of serpins requires their structural flexibility and conformational rearrangement. In order to obtain higher stability, the structures of proteins from thermophiles are usually more rigid than their mesophilic counterpart [28,29,30]. This evolution was probably driven by adaptation to the growth environments of their source organisms. Pnserpin may also acquire high stability at the cost of flexibility, and, therefore, its inhibitory activities for proteases are lower at low temperatures. The flexibility increases with temperature, and, therefore, the inhibitory activity increases as the temperature increases. Cabrita et al. [20] have reported that the disulfide bond formed by Cys102 and Cys136 was important for the stability of aeropin in extreme environments. According to the multiple sequence alignment, Pnserpin also has corresponding cysteine residues (Cys104 and Cys137) (Figure 1). In our model of Pnserpin, there is also a disulfide bond formed by Cys104 and Cys137, which may contribute to the stability of Pnserpin.

Another parameter that describes the efficiency of serpins as protease inhibitors is the *k*_ass_ of covalent complex formations. The *k*_ass_ values of Pnserpin for chymotrypsin, subtilisin, elastase, proteinase K and PnCHT are 2.46 × 10^4^, 1.30 × 10^5^, 2.27 × 10^4^, 1.98 × 10^5^, and 1.07 × 10^5^ M^−1^·s^−1^, respectively. These *k*_ass_ values are comparable with those reported for other thermophilic serpins, which range from 10^4^–10^5^ M^−1^·s^−1^ [15,17,19,20]. In summary, Pnserpin is a functional serine protease inhibitor with high temperature and pH stabilities. Many serpin genes have been found in thermophiles, but only very few of them have been biochemically and structurally characterized. Our results will help to broaden the current understanding of the inhibitory and physiological characteristics of serpins from thermophiles.

## 4. Materials and Methods

### 4.1. Cloning, Expression and Purification of Pnserpin and PnCHT Genes in E. coli

The genes of Pnserpin and PnCHT were synthesized by Generay Biotech (Shanghai Generay Biotech Co., Ltd., Shanghai, China). The full length gene of Pnserpin or PnCHT was cloned into the vector pET-28a(+), and transformed into *E. coli* BL21 (DE3) CodonPlus. The transformed cells were cultured in LB medium and 1 mM isopropyl 1-thio-β-d-galactopyranoside (IPTG) was used to induce protein expression. Cells were harvested by centrifugation at 5000 rpm for 20 min. Cell pellets were resuspended in 50 mM Tris-HCl (pH 8.0) and homogenized by ultrasonic treatment for 5 min in 3 s on/5 s off cycles, on ice. The mixture was centrifuged for 20 min at 8000 rpm at 4 °C to separate the supernatant and precipitate. The supernatant soluble protein was purified by nickel-chelating chromatography.

### 4.2. Stoichiometry of Inhibition

The stoichiometry of inhibition (SI) values of Pnserpin for proteases were determined by incubating constant amounts of proteases with increasing concentrations of Pnserpin and measuring the residual enzyme activity, as described previously [31]. Briefly, proteases at concentrations of 50 nM (CHT, elastase, PRK, thrombin, PnCHT) or 30 nM (SUC) or 10 nM (trypsin) were mixed with increasing concentrations of Pn-serpin in a sealed tube to yield molar ratios of inhibitor:enzyme (I/E) ranging from 0 to 35. The buffer contains 100 mM HEPES-NaOH (pH 7.5), 200 mM NaCl, 10 mM CaCl_2_, 0.01% (*v*/*v*) triton X-100, and 0.1% (*v*/*v*) glycerol (buffer B). The incubation times varied for different proteases depending on their stabilities as follows: 60 min at 20 and 30 °C, 30 min at 40 °C, and 2 min at 50 °C for CHT, thrombin and trypsin; 60 min at 20, 30, and 40 °C, 15 min at 50 °C, 1 min at 60 and 70 °C for SUC, elastase, and PRK; 60 min at 20, 40, and 60 °C, 30 min at 80 °C, 1 min at 100 °C for PnCHT. Samples were cooled at −20 °C for 10 min after incubation, and the residual activities of the proteases were determined in buffer B at 25 °C (40 °C for PnCHT) at 410 nm using 1 mM Succiny-Ala-Ala-Pro-Phe-*p*NA (Suc-AAPF-*p*NA) or methoxysuccinyl-Ala-Ala-Pro-Val-*p*NA (MeO-SucAAPV-*p*NA) or D-Val-Leu-Arg *p*-nitroanilidediacetate salt as a substrate, which resulted in the release of *p*-nitroaniline. A plot of the residual activity of protease as a function of the molar ratio of Pnserpin:protease yields a linear line, and the value where the line crosses the x axis was considered to be the SI value.

### 4.3. Kinetics of Inhibition

The kinetic parameters of Pnserpin for inhibition of proteases, such as the pseudo-first-order association rate constant (*k*_obs_) and second-order association rate constant (*k*_ass_), were determined using the progress curve method [32,33] at 40 or 80 °C for proteases. The concentration of Suc-AAPF-*p*NA or MeO-SucAAPV-*p*NA was 1 mM for proteases. The progress curve was obtained by monitoring the increase in absorption at 410 nm, which resulted from a release of *p*-nitroaniline, at 40 and 80 °C for PnCHT and 40 °C for other proteases. The pseudo-first-order association rate constant, *k*_obs_, was determined by non-linear regression fitting of the progress curve using Equation (1),

(1) $$P=v_{0}/k_{\mathrm{obs}}\times \left(1-e^{-k_{\mathrm{obs}}t}\right)$$

where *P* is the amount of product formation, *v*_0_ is the initial velocity, and t is the reaction time. The apparent second-order association rate constant, *k*_unc_, was determined from the slope of the *k*_obs_ value versus Pnserpin concentration (*k*_unc_ = ∆*k*_obs_/∆[I]). Because the inhibitor and substrate are competitive, the resultant *k*_unc_ value was corrected for substrate concentration ([*S*]) and Michaelis constant (*K*_m_) of the protease to calculate the second-order association rate constant, *k*_ass_, using the equation: *k*_ass_ = *k*_unc_ × (1 + [*S*]/*K*_M_). The *K*_m_ values of the proteases were determined to be 0.05 mM for CHT, 0.93 mM for SUC, 3.6 mM for elastase, 0.26 mM for PRK, and 2.85 mM for PnCHT at 40 °C, and 0.82 mM for PnCHT at 80 °C, in buffer B from Line weaver-Burk plots.

### 4.4. Temperature and pH Stabilities of Pnserpin

The Pnserpin protein was incubated at the indicated temperature for 15 min, or at the indicated pH for 24 h at room temperature. The concentration of Suc-AAPF-*p*NAor MeO-SucAAPV-*p*NA was 1 mM for proteases. The reaction was carried out in buffer B in 96-well plates. Inhibitory activity was measured at room temperature, using a BioTek microplate reader. The reaction rate in the absence of the Pnserpin was defined as 100%.

### 4.5. SDS-PAGE Analysis of the Interaction between Pnserpinand Target Proteases

Pnserpin was incubated with protease in buffer B at different molar ratios at 50 °C (80 °C for PnCHT) for 2 min. The reaction between Pnserpin and protease was stopped by the addition of reducing SDS-PAGE buffer followed by boiling for 10 min. The reaction mixture was then subjected to SDS-PAGE using a 10% polyacrylamide gel, followed by staining with 0.1% Coomassie Brilliant Blue R-250.

### 4.6. Homology Modeling and Validation of the Model Structure

The amino acid sequence of Pnserpin was collected from Genebank (accession no.ACB40836.1), in which 393 amino acid residues were involved. Two structures were identified as homologous from Protein Data Bank (PDB) (PDB ID: 2PEE, 3NDA). The automated sequence alignment (Figure 1) and analysis of the templates and target were carried out using Discovery Studio 3.5 (DS3.5). The Build Homology Models module of DS was used to create the 3D structure of Pnserpin. Out of 10 models generated, the one with the best Profile_3D profile was subjected to energy minimization. Using CHARMm force field [34], and CHARM-all-atom charges, a steepest descent algorithm was initially used to remove close van der Waals contacts, followed by conjugate gradient minimization until the energy was stable in sequential repetitions. During these steps, the quality of the initial model was improved. Profile_3D was used to check the residue profiles of the 3D models obtained. PROCHECK analysis was performed to assess the stereo-chemical qualities of the 3D models.

### 4.7. Molecular Dynamics Simulation

In order to simulate a physiological environment, physiological saline was added to Pnserpin with an explicit periodic boundary model. Then, the system was subjected to the CHARMm force-field. The protein atoms were energy-minimized by applying 1000 steps of steepest descent and 1000 steps of conjugated gradient. The temperature of the system was slowly driven from 50 to 300 K for 2 ns and equilibration simulations were run for 1 ns. The production were performed for 10 ns at a constant temperature of 300 K and a constant pressure, and the results were saved at a frequency of 0.02 ns. The MD trajectory was determined by using the DS3.5 analyze trajectory protocol.

## Figures and Tables

**Table 1:** Stoichiometry of inhibition (SI) values of Pnserpin for serine proteases.

Temperature								
Enzyme	20 °C	30 °C	40 °C	50 °C	60 °C	70 °C	80 °C	100 °C
CHT	9.49	5.80	4.76	2.21	- ^a^	- ^a^	- ^a^	- ^a^
SUC	10.04	8.76	6.95	5.34	3.97	2.67	- ^a^	- ^a^
elastase	10.06	9.33	7.9	7.41	4.84	2.74	- ^a^	- ^a^
PRK	9.99	7.39	5.99	4.69	2.75	2.28	- ^a^	- ^a^
thrombin	21.13	15.16	12.22	9.31	- ^a^	- ^a^	- ^a^	- ^a^
trypsin	10.19	6.14	5.16	2.53	- ^a^	- ^a^	- ^a^	- ^a^
PnCHT	32.72	- ^a^	23.92	- ^a^	19.83	- ^a^	11.26	6.82

^a^ The data was undetected.

**Table 2:** The results of PROCHECK and Profile_3D verification.

Program	Results	
PROCHECK	Residues in most favored regions	92.3%
	Residues in additional allowed regions	6.4%
	Residues in generously allowed regions	1.2%
	Residues in disallowed regions	0%
Profile_3D	Verify score	169.8
	Verify expected high score	170.9
	Verify expected low score	76.9

## References

[1] Irving J.A., Pike R.N., Lesk A.M., Whisstock J.C. (2000). Phylogeny of the serpin superfamily: Implications of patterns of amino acid conservation for structure and function. Genome Res..

[2] Law R.H., Zhang Q., McGowan S., Buckle A.M., Silverman G.A., Wong W., Rosado C.J., Langendorf C.G., Pike R.N., Bird P.I. (2006). An overview of the serpin superfamily. Genome Biol..

[3] Rodgers G.M. (2009). Role of antithrombin concentrate in treatment of hereditary antithrombin deficiency. An update. Thromb. Haemost..

[4] Hunt J.M., Tuder R. (2012). α 1 anti-trypsin: One protein, many functions. Curr. Mol. Med..

[5] Janciauskiene S.M., Bals R., Koczulla R., Vogelmeier C., Köhnlein T., Welte T. (2011). The discovery of α1-antitrypsin and its role in health and disease. Respir. Med..

[6] Ma J., Tong Y., Yu D., Mao M. (2012). Tissue plasminogen activator-independent roles of neuroserpin in the central nervous system. Neural Regen. Res..

[7] Yepes M., Lawrence D.A. (2004). Neuroserpin: A selective inhibitor of tissue-type plasminogen activator in the central nervous system. Thromb. Haemost..

[8] Zheng D., Chen H., Davids J., Bryant M., Lucas A. (2013). Serpins for diagnosis and therapy in cancer. Cardiovasc. Hematol. Disord. Drug Targets.

[9] Jaadane I., Nagbou A., Behar-Cohen F., Torriglia A. (2014). Interaction of Leukocyte Elastase Inhibitor/L-DNase II with BCL-2 and BAX. Biochim. Biophys. Acta.

[10] Morito D., Nagata K. (2012). ER Stress Proteins in Autoimmune and Inflammatory Diseases. Front. Immunol..

[11] Huntington J.A. (2011). Serpin structure, function and dysfunction. J. Thromb. Haemost..

[12] Devlin G.L., Bottomley S.P. (2005). A protein family under “stress”—Serpin stability, folding and misfolding. Front. Biosci..

[13] Whisstock J.C., Bottomley S.P. (2006). Molecular gymnastics: Serpin structure, folding and misfolding. Curr. Opin. Struct. Biol..

[14] Cho Y.L., Chae Y.K., Jung C.H., Kim M.J., Na Y.R., Kim Y.H., Kang S.J., Im H. (2005). The native metastability and misfolding of serine protease inhibitors. Protein Pept. Lett..

[15] Irving J.A., Cabrita L.D., Rossjohn J., Pike R.N., Bottomley S.P., Whisstock J.C. (2003). The 1.5 Å crystal structure of a prokaryote serpin: Controlling conformational change in a heated environment. Structure.

[16] Fulton K.F., Buckle A.M., Cabrita L.D., Irving J.A., Butcher R.E., Smith I., Reeve S., Lesk A.M., Bottomley S.P., Rossjohn J. (2005). The high resolution crystal structure of a native thermostable serpin reveals the complex mechanism underpinning the stressed to relaxed transition. J. Biol. Chem..

[17] Zhang Q., Buckle A.M., Law R.H., Pearce M.C., Cabrita L.D., Lloyd G.J., Irving J.A., Smith A.I., Ruzyla K., Rossjohn J. (2007). The N terminus of the serpin, tengpin, functions to trap the metastable native state. EMBO Rep..

[18] Zhang Q., Law R.H., Bottomley S.P., Whisstock J.C., Buckle A.M. (2008). A structural basis for loop C-sheet polymerization in serpins. J. Mol. Biol..

[19] Tanaka S., Koga Y., Takano K., Kanay S. (2011). Inhibition of chymotrypsin- and subtilisin-like serine proteases with Tk-serpin from hyperthermophilicarchaeon *Thermococcus kodakaraensis*. Biochim. Biophys. Acta.

[20] Cabrita L.D., Irving J.A., Pearce M.C., Whisstock J.C., Bottomley S.P. (2007). Aeropin from the extremophile *Pyrobaculum aerophilum* bypasses the serpin misfolding trap. J. Biol. Chem..

[21] Chan P.P., Cozen A.E., Lowe T.M. (2013). Reclassification of *Thermoproteus neutrophilus* Stetter and Zillig 1989 as *Pyrobaculum neutrophilum* comb. nov. based on phylogenetic analysis. Int. J. Syst. Evol. Microbiol..

[22] Hopkins P.C., Carrel R.W., Stone S.R. (1993). Effects of mutations in the hinge region of serpins. Biochemistry.

[23] Robert X., Gouet P. (2014). Deciphering key features in protein structures with the new END script server. Nucleic Acids Res..

[24] Chmelar J., Oliveira C.J., Rezacova P., Francischetti I.M., Kovarova Z., Pejler G., Kopacek P., Ribeiro J.M., Mares M., Kopecky J. (2011). A tick salivary protein targets cathepsin G and chymase and inhibits host inflammation and platelet aggregation. Blood.

[25] Laskowski R.A., MacArthur M.W., Moss D.S., Thorntonl J.M. (1993). PROCHECK: A program to check the stereochemical quality of protein structures. J. Appl. Crystallogr..

[26] Luthy R., Bowie J.U., Eisenberg D. (1992). Assessment of protein models with three-dimensional profiles. Nature.

[27] (1999). Profile-3D User Guide.

[28] Jaenicke R. (2000). Do ultrastable proteins from hyperthermophiles have high or low conformational rigidity?. Proc. Natl. Acad. Sci. USA.

[29] Fields P.A. (2001). Protein function at thermal extremes: Balancing stability and flexibility. Comp. Biochem. Physiol. A Mol. Integr. Physiol..

[30] Radestock S., Gohlke H. (2008). Exploiting the link between protein rigidity and thermostability for data-driven protein engineering. Eng. Life Sci..

[31] Ksiazek M., Mizgalska D., Enghild J.J., Scavenius C., Thogersen I.B., Potempa J. (2015). Miropin, a novel bacterial serpin from the Periodontopathogen *Tannerella forsythia*, inhibits a broad range of proteases by using different peptide bonds within the reactive center loop. J. Biol. Chem..

[32] Schechter N.M., Plotnick M.I. (2004). Measurement of the kinetic parameters mediating protease-serpin inhibition. Methods.

[33] Morrison J.F., Walsh C.T. (1988). The behavior and significance of slow-binding enzyme inhibitors. Adv. Enzymol. Relat. Areas Mol. Biol..

[34] Brooks B.R., Bruccoleri R.E., Olafson B.D., States D.J., Swaminathan S., Karplus M. (1983). CHARMM: A program for macromolecular energy, minimization, and dynamics calculations. J. Comp. Chem..

